# The Resulting Effect of Flow Pulsations on Calibration Constant of Acoustic Path in Ultrasonic Flowmeters

**DOI:** 10.3390/s22072815

**Published:** 2022-04-06

**Authors:** Anna Goltsman, Ilya Saushin

**Affiliations:** FRC Kazan Scientific Center, Institute of Power Engineering and Advanced Technologies, Russian Academy of Sciences, 2/31 Lobachevsky St., P.O. Box 261, 420111 Kazan, Tatarstan, Russia; ilyasaushin@mail.ru

**Keywords:** flow measurement, pulsating flow regimes, ultrasonic flowmeter, flow rate, pulsating flow

## Abstract

The present paper compares, for the first time, the regimes of a pulsating turbulent flow in a smooth pipe in terms of 0.001 ≤ ω^+^ ≤ 0.0346 and 0.16 ≤ β ≤ 0.63 at Re ≈ 7000 with the uncertainty in estimating the flow rate by an ultrasonic flowmeter. It was revealed that the classification of pulsating flow regimes according to the dimensionless angular frequency ω^+^ does not have a direct relation with the K parameter equal to the ratio of the phase-average calibration constant in pulsating flow to the corresponding value in steady flow. The results of data processing showed that K depends on the relative amplitude of pulsations β and the position of the chord of the ultrasonic beam trajectory (L/R is distance L from the pipe center to the chord to the pipe radius R). In the coordinates β and L/R, there is a rather wide area where the uncertainty in flow rate estimation of pulsating flows should not exceed 0.5%. An increase in β or L/R leads to an increase in measurement uncertainty, which in the limiting case β, L/R → 1 can reach 5% or more. Favorable and unfavorable areas of the pipe section were identified when scanning pulsating flows and the effectiveness of using multi-path scanning schemes was estimated to reduce the resulting effect of flow pulsations on flow measurement uncertainty.

## 1. Introduction

The operation principle of an ultrasonic flowmeter is to measure the transit time of acoustic oscillations through a liquid or gas flow, which depends on the flow velocity. Flow measurement of continuous media by ultrasound has many scientific and technical challenges [1], ranging from piezoelectric sensor design to signal processing techniques. The present paper considers only those problems that may become relevant when measuring pulsating flows, namely we focus only on the resulting effects of the deformed profile of the pulsating flow velocity.

Accurate measurements with ultrasonic flowmeters are associated with the nature of the flow in front of the measurement section. In practice, in most cases, measurements are carried out precisely in round pipes, so the main difficulty is the implementation of the conditions for a developed axisymmetric or uniform flow velocity profile in front of the flowmeter. This makes it possible to operate with one pair of senders located in one diametrical plane, for example. The only drawback, specifically, for a developed pipe flow is that the measured averaged over any chord of the pipe section flow velocity almost never coincides with the average flow velocity. An exception to this rule is for a chord located at a distance of (0.5–0.54) R from the center of the pipe, where R is the radius of the pipe. However, measurements on this chord are usually used only for refinement measurements in multipath flowmeters. The problem of the inequality of the measured velocity on the ray trajectory of the average flow velocity nevertheless resolves with certain difficulties by means of preliminary experimental calibration of the device or estimation of the correction factor with sufficient accuracy. There are also devices for preparing the flow in front of the measurement section which are able to adjust this correction factor to be close to unity. Such devices include, for example, the contraction section (convergent channel).

However, in many cases, especially when it comes to pipes with large diameters, it is very difficult to provide a straight section of sufficient length (from 50 D for asymmetric and up to 200 D for swirl profiles [2], where D is the pipe diameter) in front of the flowmeter to form a developed axisymmetric profile. Measuring the flow rate under conditions of a deformed velocity profile is the second problem. There are two main technical solutions here: the installation of a flow conditioner and/or the measurement of velocity along several chords of the pipeline cross-section. Installation of a flow conditioner significantly reduces the required length of the straight section of the pipeline in front of the flowmeter but significantly increases hydraulic losses. Scanning a multi-chord, asymmetrical velocity profile, of course, implies the use of a multi-path flowmeter, which in turn is more expensive than a single-path or dual-path flowmeter.

Nevertheless, it can be said that the areas of flow measurement with ultrasonic flowmeters of steady pipe flows are known and that all the main problems have been investigated and acceptable methods for their solution have been developed.

However, in practice, there are also the unsteady periodic pulsating flows of a continuous medium in hydraulic systems for the transporting of gas or liquid by compressor systems, resonant pipe vibrations, flow-control valves, the phenomenon of flow separation behind the obstacle in pipelines, and some multiphase flow regimes that are present in industry (energy, chemical, automotive, pharmaceutical, and food industries) [3,4,5,6].

By itself, the theory of pulsating pipe flows is a separate and rather complex area of continuum mechanics. In contrast to steady flows, in an unsteady pulsating flow, according to the theory of dimensions, there should be two more dimensionless parameters, in addition to the Reynolds number (Re), describing the frequency and amplitude of pulsations. Moreover, if everything is more or less clear with the amplitude characteristic (usually the dimensionless parameter β is used—the ratio of the amplitude of the pulsations to the value of the average velocity over the period), then with the frequency characteristic everything is not so evident. As the theory of pulsating flows developed, attempts were made to use the Womersley number [7], the Strouhal number [8,9], and the dimensionless angular frequency ω^+^ = ων/u_τ_^2^ (where ω = 2 π/T is the angular frequency, T is the period of the pulsations, ν is the kinematic viscosity, and u_τ_ is the dynamic velocity) [10,11,12,13]. Based on the experimental, numerical and theoretical studies [9,11,13,14,15,16,17,18,19,20,21,22,23,24] today it is usual to distinguish five regimes of turbulent pulsating flow in a smooth channel:

- ω^+^ → 0. *Quasi-steady regime*. Such unsteady flows can be described as a successive change in steady turbulent flows without the effect of the flow history. In this case, changes in the values of the flow parameters occur without a phase lag. The processes occurring in these flows are well described by steady methods.
- ω^+^ < 0.005. *Low-frequency regime*. The effect of the pulsations is spread throughout the boundary layer thickness. The phases of the velocity pulsations in the flow core and shear stresses on the wall do not already coincide.
- 0.005 < ω^+^ < 0.02. *Intermediate-frequency regime*. A region of frozen turbulence located in the flow core appears with the nature of the piston flow movement.
- 0.02 < ω^+^ < 0.04. *High-frequency regime*. This regime is characterized by a significant effect of pulsations only within the viscous sublayer. The flow outside this area moves like a solid mass with freezing turbulence. The lags in the velocity pulsations in the flow core and shear stresses correspond to the Stokes law for laminar flows.
- 0.04 < ω^+^. *Very-high-frequency regime*. The frequency of flow pulsations is comparable to the frequency of turbulent pulsations of a steady flow.

When measuring the flow rate of pulsating flows with a flowmeter, this means that the profile of the averaged, developed flow can depend on the frequency–harmonic characteristics of the unsteadiness of the periodic imposed flow at the same flow rate of the medium. It is an accepted fact that the instantaneous and even the phase-averaged velocity profile of the laminar and turbulent pulsating flow in the pipe can have an M-shape practically in any experimental work on the study of pulsating flows in a pipe [11,14,25,26,27]. Naturally, this fact contributed to the appearance of papers where the resulting effect of the presence of the pulsations on the reliability of flow measurements by various types of flowmeters was estimated.

The flow rate of a continuous medium in a pulsating flow was measured by almost all types of existing flowmeters: Venturi [28], Pitot tube [28,29], with a film element [28], vortex [1,28], turbine [28,30,31], current meters ([32] Jepson 1967), differential [27,33], and cross-correlation [1]. Generalization of these results (but only based on the pulsating flow regimes investigated by the authors) allows drawing the following conclusion: the measurement uncertainty increases significantly with an increase in the relative amplitude of pulsations, and to a greater extent this is aggravated in the designs of flowmeters with elements in contact with the pulsating flow [34]. For example, a turbine flowmeter responds more quickly to an increase in flow rate than to a decrease, which leads to an overestimation of the average flow [30], especially when measuring gas flow rates [31]. Therefore, as applied to pulsating flows, non-contact flow measurement methods, such as an ultrasonic flowmeter, are of particular interest.

By their structure, the developed, pulsating flows in the pipe are regarded as steady, but, for example, they were originally excluded from the normal scope of the standards for measuring the flow of liquids and gases by means of pressure-differential devices inserted in circular cross-section conduits running full [33,35,36]. Subsequently, additional regulatory documents were issued for measuring the flow rate of pulsating flows [37,38]. In particular, the introduction of appropriate corrections to the readings of the flowmeter or the calculation of the estimation of an additional component of the measurement uncertainty of the medium amount were proposed. However, even with these additions, there are restrictions on the permissible relative root-mean-square amplitude of the mean-frequency pulsations; therefore, not all regimes of pulsating flows are subject to the standards. As for the standard on flow measurement using ultrasonic transducers [2], it proposes the use of calibration constants only when the Reynolds number, physical properties of the continuum, and the thermal deformations of the body change. However, [2] it does not specify the possibility of using such amendments in the case of pulsating flows. It is assumed in [2] that ultrasonic flowmeters should simply “deal with” pulsating flows well, and the meter manufacturer should specify the allowable frequency–harmonic flow parameters in the instrument specifications. A reduction in the measurement uncertainty of pulsating flow rate can be ensured, for example, by acoustic signals fired at a non-constant rate.

Our experience on the study of pulsating flows [26,39] has allowed us to investigate the estimation of the reliability of flow measurement by ultrasonic flowmeters with a great emphasis on the flow structure under known pulsating flow regimes. Other authors varied the frequency and amplitude of pulsations without an important comparison with the map of pulsating flow regimes, which precisely determines the unsteady hydrodynamic flow structure. The present paper shows, for the first time, an estimation of the resulting effect of the frequency–harmonic characteristics of pulsating flows on the calculated value of the k calibration constant. This constant describes the ratio of average flow rate to the average velocity on the trajectory of acoustic disturbances for basic acoustic path types for single- and multi-path meters [2]. We also determined favorable and unfavorable areas of the pipe section for the location of the paths of ultrasonic beams. The results of the study make it possible to preliminarily estimate the uncertainty in measuring the flow rate of pulsating flows in a wide range of frequency–harmonic characteristics depending on the flow-scanning method.

## 2. Materials and Methods

### Basic Acoustic Path Types and Calibration Constant

**Figure 1:**
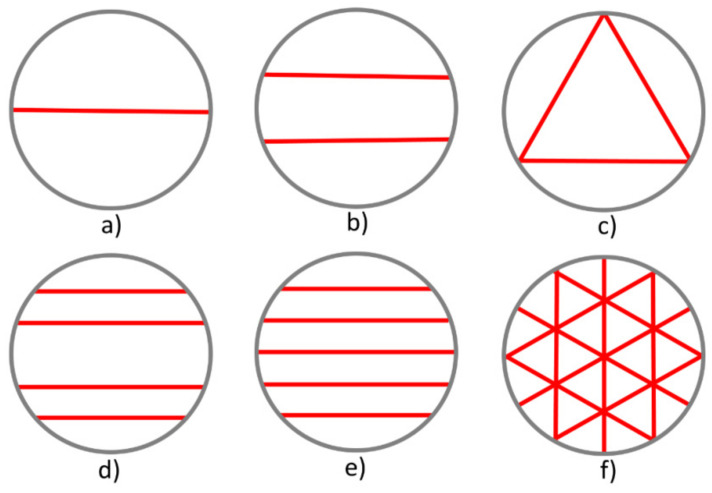
Basic acoustic path types for single- and multi-path meters: (**a**) one diametric; (**b**) two parallel one-third diametric; (**c**) three mid-radius; (**d**) four parallel; (**e**) five parallel; (**f**) mid-radius and diametric.

Several schemes for acoustic signal trajectories for single and multi-path flowmeters are proposed in [2]. These trajectories can be divided into six types according to the number and location of beam trajectories, as shown in Figure 1 and Table 1. For easy identification, each chord is further identified by L value. L is the distance from chords to the section center. In schemes (b) and (c), the flow is scanned along equivalent chords (Figure 1); therefore, these schemes are considered as single-path in the case of symmetric velocity profiles in the present paper.

Due to the effect of viscosity forces in the near-wall region, the profile of the turbulent flow velocity in a round pipe is not uniform. Therefore, the value of average velocity along any of the chords is not equal to the average flow rate (except in an accidental coincidence). For a steady developed flow, the ratio of these velocities is determined by the Re number. ISO [2] recommends the use of the k (Re, L) calibration constant to reconstruct the true flow rate from chord velocity measurements. The *k* calibration constant is calculated as the ratio of the nominal flow rate *Q* to the flow rate obtained as the product of the measured average flow rate *U_L_* on the beam path and the flow area *F*, relation (1):

(1) $$k=\frac{Q}{U_{L}\cdot F}.$$

For the follow-up estimation of the resulting effect of flow pulsations, we determined the dependence k (L), where k is the calibration constant for any of the chords in the section. We chose a flow regime at Re = 7000, at which the flow was turbulent, and the shape of the velocity profile was farthest from uniform.

To estimate the dependence of the calibration constant on the Re number, we used the results of the numerical simulation of a steady water flow in a 50.8 mm diameter and 6600 mm long, round pipe at Re = 7000, which corresponded to the experimental setup [11]. The calculations were performed using the ANSYS Fluent 19.2 software (ANSYS Academic Research CFD: 1 task Permanent with TECS expiring 29-Nov-2019 Customer # 1075016, Russia, Kazan).

Given the triviality of this simulation, we only briefly describe the numerical problem statement. The motion of a continuous medium (water) was described by Reynolds-averaged Navier-Stokes equations with isotropic Shear-Stress Transport k-ω model with Low-Re Correction option. Pressure was interpolated using a second-order scheme, pressure-velocity coupling was achieved by the semi-implicit method for pressure-linked equations (SIMPLE) algorithm, the second-order scheme was employed to estimate moments, and turbulence parameters were approximated by a second-order upwind differencing scheme. The 3D geometric model was identical to the experimental setup [11], except that the uniformity of the velocity profile at the channel inlet was provided by the boundary condition. The boundary condition set in the inlet was water’s constant velocity (0.138 m/s) with a constant density of 998.2 kg/m^3^ and dynamic viscosity of 0.001003 Pa·s. Static pressure of 0 Pa was specified in the outlet. Residual values of iterative solution and gas flow rate balance between inlet and outlet were taken as convergence criteria. The hexahedral mesh was generated in ANSYS Meshing. During the calculation, the mesh was adapted in ANSYS Fluent to achieve a maximum size of the wall cells of no more than 1y^+^. After adaptation, the number of calculated cells was about 3.3 million.

**Figure 2:**
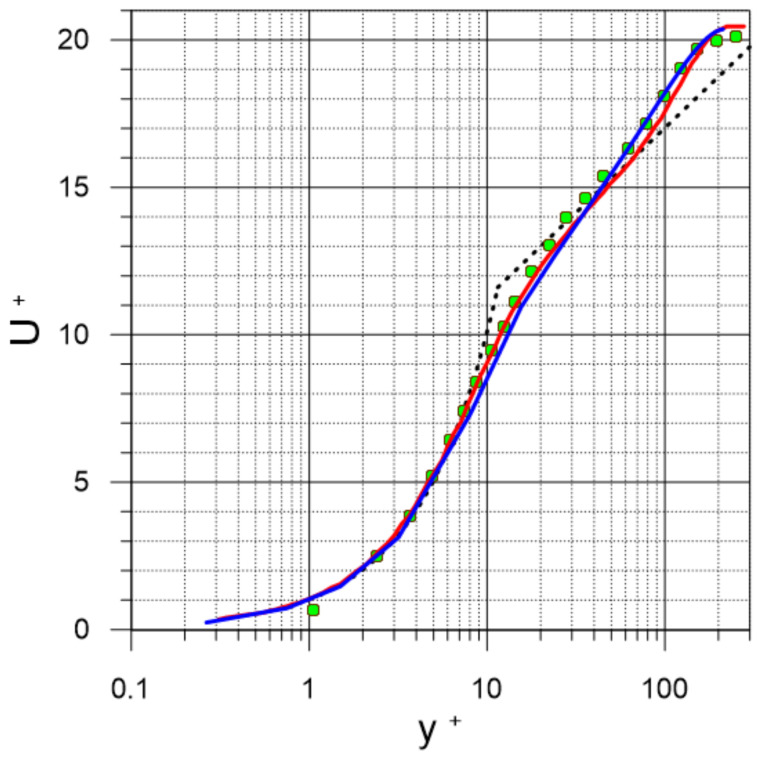
U^+^ (y^+^) velocity profile in the boundary layer of turbulent flow; black dashed line = logarithmic law; solid red line = DNS (Re_θ_ = 590; [40]); green circles = PIV (Re_θ_ = 518; [41]); solid blue line = RANS (Re_θ_ = 490; this work).

Figure 2 shows our RANS results for the velocity profile (U^+^ = U/u_τ_) in y^+^ = yu_τ_/ν coordinates; RANS results are in good agreement with the results of direct numerical simulation [40] and the experimental measurements by the Particle Image Velocimetry (PIV) method [41] at close values Re_θ_ = θU_∞_/ν (where u_τ_ is the dynamic velocity, θ is the momentum thickness, and ν is the kinematic viscosity). The larger angle of inclination in the logarithmic section of the velocity profile obtained by DNS and PIV is associated with the presence of a streamwise pressure gradient (characteristic for channel flows), while the theoretical profile (line) was obtained under conditions of free flow around the plate.

**Figure 3:**
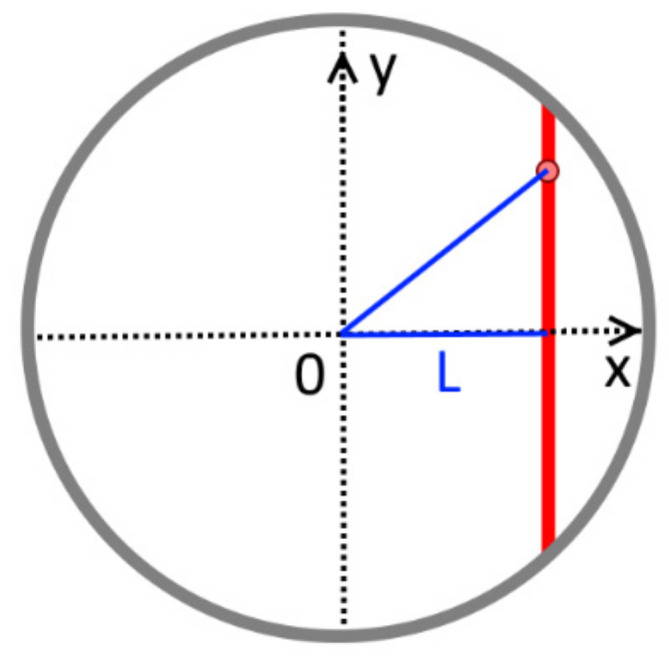
Obtaining the velocity profile of chord.

Since we are considering a developed symmetric flow profile, the velocity is a function only of the pipe radius. To obtain the function *k* (*L*), the velocity profile on any of the chords was expressed in terms of a 10th degree polynomial (2):

(2) $$U_{L}\left(y\right)={\sum_{i=0}^{10}a_{i}\left(\sqrt{y^{2}+L^{2}}\right)}^{i},$$

where *a_i_* are polynomial coefficients, *L* is the distance from the chord to the center of pipe section, and *y* is the point coordinate, as shown in Figure 3.

**Figure 4:**
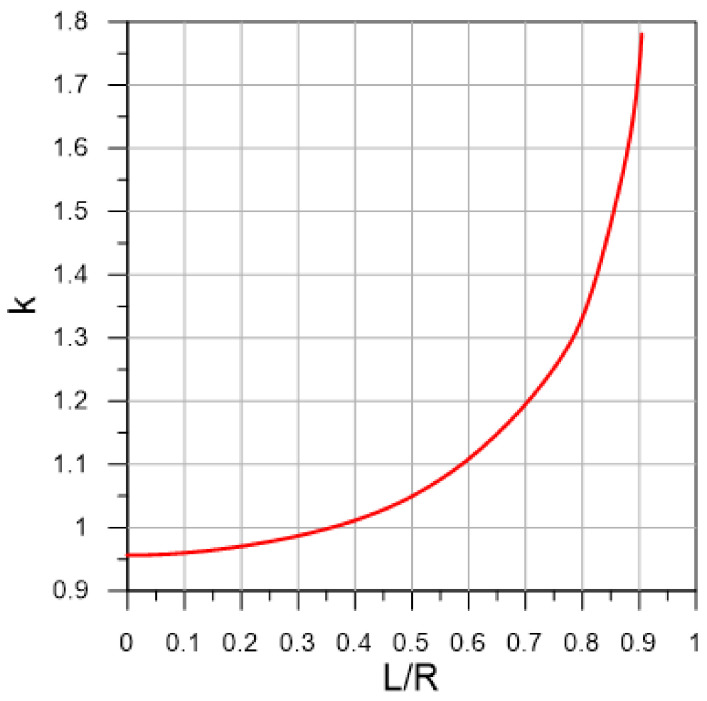
Dependence of the calibration constant on the distance L.

Figure 4 shows the dependence of the calibration constant on the distance L obtained from expressions (1) and (2).

## 3. Results

### 3.1. Uncertainty Estimation in Measuring of the Pulsating Flow Rate in a Circular Pipe

#### 3.1.1. Dynamics of Velocity Profiles in Pulsating Flow

The first I quasi-steady regime is characterized by flow that can be described as a successive change of steady turbulent flows; therefore the other four regimes (II–V) are of the greatest interest. The range in the region of Re ≈ 7000 is especially interesting, where the pipe flow is still far from the self-similar state with respect to the change in Re. In previous work [42], based on the results of experimental and numerical studies, it was shown that the calibration constant function k (Re) has the greatest gradient in the above range, which is an additional motivation for studying this particular interval as potentially the worst one for measuring the pulsating flow rate.

Velocity profiles along the entire height of the pipe diameter for pulsating flow regimes II–V can be found, for example, in the experimental work [11], in [20], where the profiles were obtained from the results of direct numerical simulation (DNS), as shown in Table 2.

**Figure 5:**
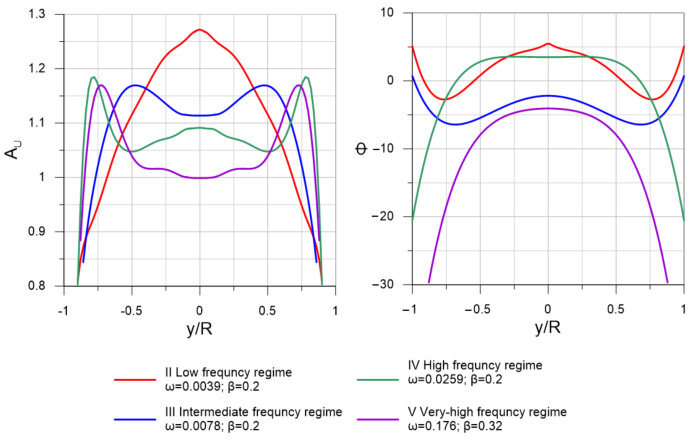
Amplitude A_U_ of velocity modulation in pulsating flows and phase shift Φ of velocity modulation relative to the imposed flow pulsation.

However, the dynamics of velocity profiles are not presented explicitly in [11,20], only the amplitudes A_U_ of velocity modulations and the phase shift Φ of velocity modulation relative to the imposed flow pulsation are shown. Figure 5 shows examples for four regimes. A laser Doppler anemometer (LDA) system was used in [11], so the velocity measurements are presented at about 12 points along the pipe radius. To obtain continuous velocity profiles, we approximated these data with 3–8 degree polynomials. The degree of the polynomial was chosen depending on the shape of the profile.

Reproduction of the velocity profiles by the phase angle 0 ≤ φ < 2π of the pulsation period for each regime from Table 2 was carried out by the relation (3):

(3) $$U\left(r\right)=\bar{U}\left(r\right)\left[1+\beta A_{U}\left(r\right)Sin\left[\varphi -\Phi \left(r\right)\right]\right],$$

where $\bar{U}\left(r\right)$ is the velocity profile of developed steady flow in a pipe. Since we simultaneously use the results of estimating the velocity profiles obtained by different methods (DNS, RANS, LDA) for the analysis, the values of the flow rate estimated by the integral ratio (4) in the Wolfram Mathematica program were compared first of all.

(4) $$Q=2\pi \int_{0}^{R}rU\left(r\right)dr.$$

Further, all the profiles of pulsating flow were brought to a single integral flow rate characterized by multiplying by a correction coefficient equal to the ratio of integral flow rate to the reference value. The flow rate obtained by integration over the steady flow velocity profile (RANS) was taken as a reference value. The correction coefficients were in 0.98 ÷ 1.025. Thus, the analyzed flow rate profiles corresponded to identical flow rates.

**Figure 6:**
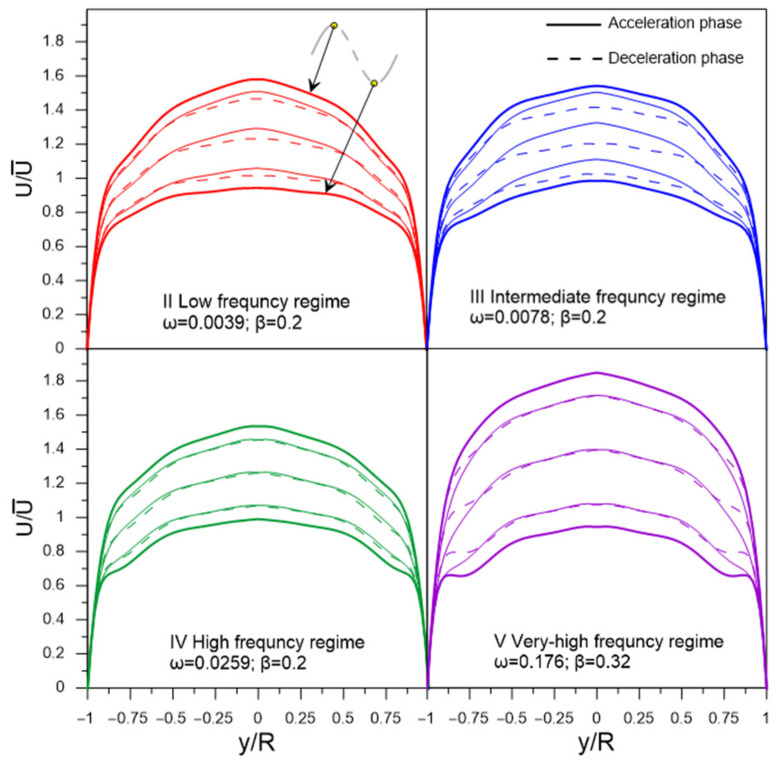
Reconstructed dynamics of velocity profiles.

Figure 6 shows the reconstructed dynamics of velocity profiles for four regimes in the phase angle with a step φ = π/4. For clarity, the acceleration and deceleration phases are indicated in the graphs.

#### 3.1.2. Effect of Flow Pulsations on the Calibration Constant on A Single Chord

It is obvious that the operation frequency of the source of ultrasonic disturbances of the device in practice almost never completely coincides with the frequency of the flow pulsations. Consequently, with a sufficient time interval for measurements, the statistical volume of measurements will completely cover the entire phase interval of the pulsation period. Therefore, to estimate the average value of the calibration constant in pulsating flow, we divide the period of pulsations into equal intervals. For example, we divide by 12 with a phase angle step φ = π/6, since the number of partitions into 12 intervals is sufficient to describe the period of a harmonic signal. Let us introduce for consideration the K parameter, which is equal to the ratio of the phase-average calibration constant in pulsating flow to the corresponding value in steady flow. The deviation of K from 1 can be identified with the value of the uncertainty in estimating the flow rate of pulsating flow, which is measured by an ultrasonic flowmeter calibrated on steady flow.

**Figure 7:**
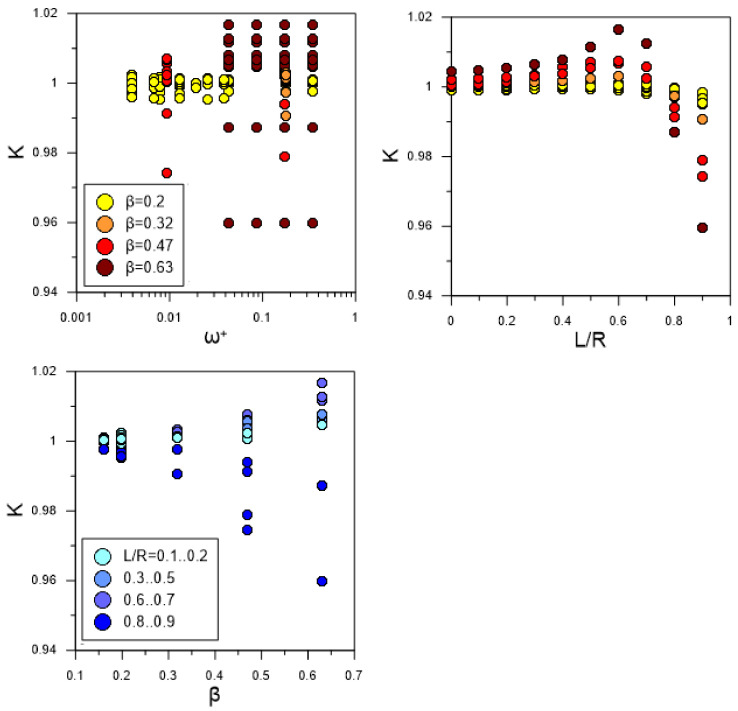
Dependence of K on the frequency–harmonic characteristics of flow and L/R.

**Figure 8:**
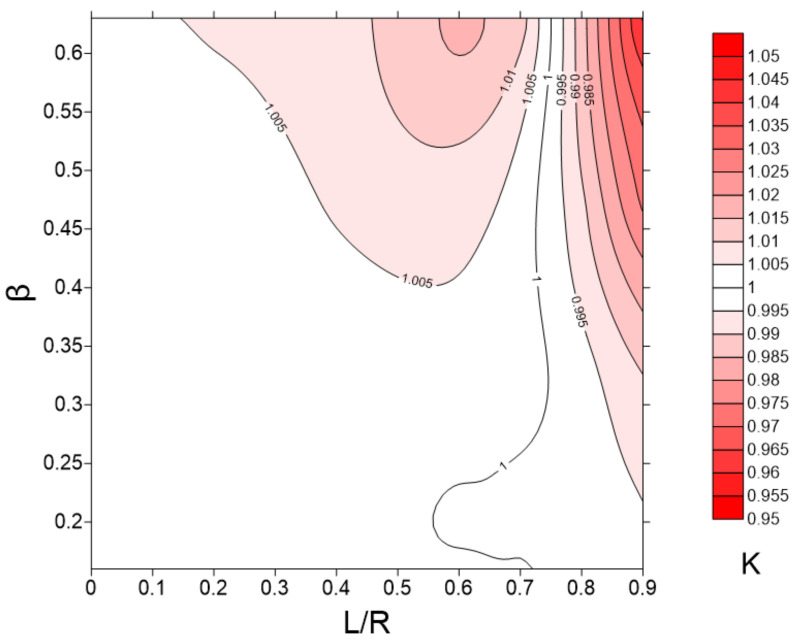
Dependence of K at the chord on the distance L to the section center and the relative amplitude of pulsations β.

**Figure 9:**
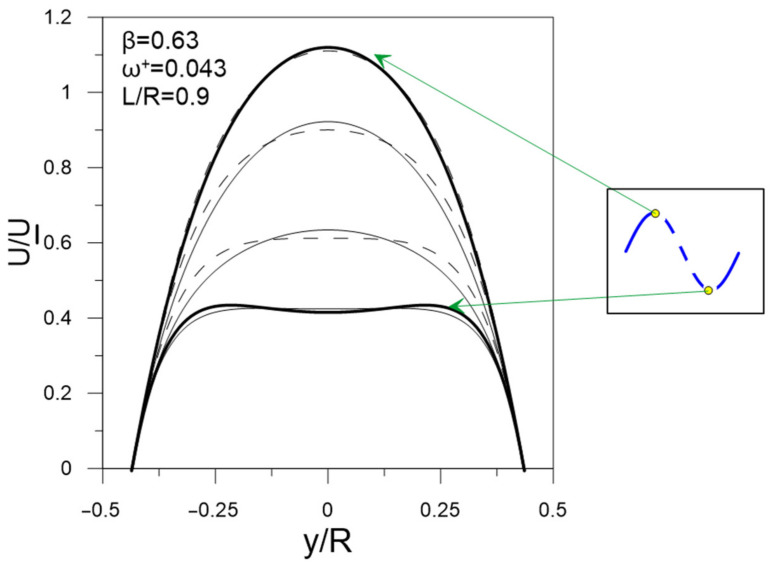
Dynamics of velocity profile on chord near the wall.

Since K can immediately depend on ω^+^, β, and L/R parameters, we first constructed graphs of the K dependence on each of three parameters as shown in Figure 7. As can be seen from the graphs, the value of K does not depend on the dimensionless angular frequency ω^+^ and depends on the relative amplitude of pulsations β and the position of the chord L/R. Therefore, it is convenient to analyze the dependence of K on the contour graph as a function of two variables, as shown in Figure 8. The results show that the location of ultrasonic beam path within a distance from the center of the section of no more than 0.4 R should lead to an uncertainty in the estimate of flow rate of no more than 1% for pulsating flows with an amplitude of up to β ≈ 0.6. The most unfavorable area of the chord location is the region L/R > 0.8, where the resulting effect of pulsations is observed even at small amplitudes of pulsations and sharply increases with further distance of the chord from the section center. This is due to the fact that the chord covers the region of large gradient of the function Φ(r) near the wall (Figure 5), which leads to a pronounced change in the shape of the velocity profile along the phase angle, as shown in Figure 9; and thus an increase in the deviation of K from 1, as shown in Figure 8. For comparison, the velocity profiles on the diametrical chord (Figure 5) almost remain similar, which leads to K values close to 1, as shown in Figure 8.

### 3.2. Uncertainty in Estimating the Flow Rate in Pulsating Flow for Different Acoustic Path Types

**Figure 10:**
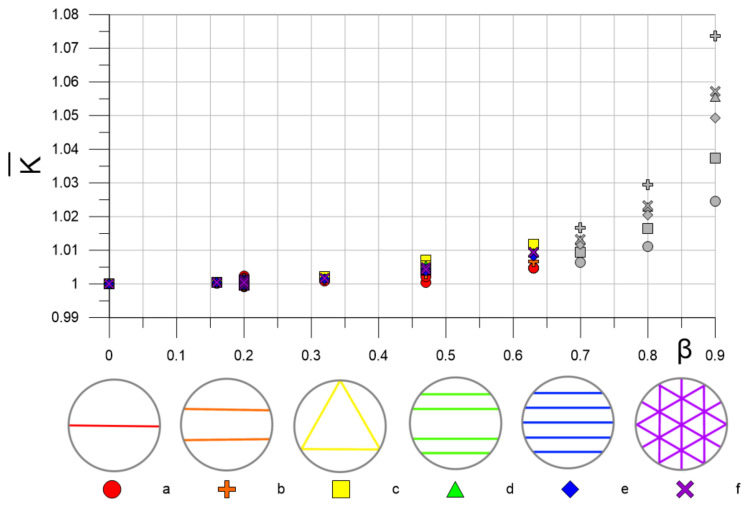
The resulting effect of flow pulsations on the averaged calibration constant for different acoustic path types. Gray markers are predictable values.

In the previous section, Figure 8 showed the resulting effect of flow pulsations on K values for single chords. In the case of a multi-path flowmeter, there are several chords, so the uncertainty of flow measurements will depend on the acoustic path scheme, which consists of several chords. By the value for acoustic path, we mean the average value of K on all chords. Figure 10 shows the dependencies $\bar{K}\left(\beta \right)$ for acoustic path types from [2], in Figure 1 and Table 1. As expected, the values $\bar{K}\left(\beta \right)$ do not exceed 1.015 for pulsating flows at β ≤ 0.63 because none of the chords in all the acoustic path types lies in the region L/R > 0.7. For pulsating flows with β < 0.3, all schemes can be considered equivalent, and the expected uncertainty in estimating the flow rate should not exceed 0.3%. The growth of the uncertainty value starts from β ≈ 0.35 to β ≈ 0.7.

We also added gray markers on the graph—this is a prediction of further growth of K with increasing β. This estimate is based on the fact that for very-high-frequency regime V the profile of the harmonic part of the pulsating flow velocity is described by the real part of the Stokes solution [13,20,43]:

(5) U˜r,t=Re[−iρωdpdx^1−J0ωνri32J0ωνRi32eiωt],

where *J*_0_ is Bessel function of the first kind, *i* is the imaginary unit, *ρ* is the density, t is the time, *dp/dx* with the circumflex is the amplitude of streamwise (*x*) pressure gradient pulsations. Therefore, it can be assumed that the amplitude *A_U_* of velocity modulation and phase shift Φ of velocity modulation relative to the imposed flow pulsation do not depend on β and *ω^+^* for the V regime.

## 4. Discussion

The present paper compares, for the first time, the regimes of a pulsating turbulent flow in a smooth pipe in terms of ω^+^ and β with the uncertainty in estimating the flow rate by an ultrasonic flowmeter.

Using the results of DNS [20] and the experimental measurements [11] of the velocity profiles in a pulsating pipe flow at various values of 0.001 ≤ ω^+^ ≤ 0.0346 and 0.16 ≤ β ≤ 0.63 at Re ≈ 7000, the predicted dependences of the measurement uncertainty of the flow rate in different acoustic path types by [2] on these parameters were considered. Consideration of almost limiting minimum Re number, at which the flow is turbulent and the velocity profile is maximally inhomogeneous, suggests that the present paper has estimated the upper limit of the uncertainty in measuring the flow rate of developed pulsating flows by ultrasonic flowmeters.

Firstly, it was revealed that the classification of pulsating flow regimes according to the dimensionless angular frequency ω^+^ does not have a direct relation with the K parameter equal to the ratio of the phase-average calibration constant in pulsating flow to the corresponding value in steady flow. The results of data processing showed that K depends on the relative amplitude of pulsations β and the position of the chord of the ultrasonic beam trajectory L/R. According to the map of the K parameter in the coordinates β and L/R (Figure 8), there is a rather wide area where the uncertainty in flow rate estimation of pulsating flows should not exceed 0.5%. An increase in β or L/R leads to an increase in the measurement uncertainty, which in the limiting case β, L/R → 1 can reach 5% or more. This is mainly due to the phenomenon of phase shift Φ of velocity modulation relative to the imposed flow pulsation, which is most pronounced in the near-wall region flow. Therefore, to measure the flow rate of pulsating flows, the condition L/R < 0.8 must be met. Since the different acoustic path types proposed by [2] satisfy this requirement, the use of these schemes for measuring the flow rate of pulsating flows with β < 0.65 should lead to an uncertainty value of no more than 1.5%. However, with an increase in β for all schemes, an almost exponential increase in the uncertainty is observed, which has a higher gradient for schemes containing chords far from the center of the section. Obviously, if the velocity profile in the measurement section is not symmetric, the efficiency of single-path schemes is likely to be significantly inferior to multi-path ones.

According to the obtained results (Figure 10), the measurement uncertainty of the flow rate of pulsating flows increases with increasing relative amplitude β. To reduce this uncertainty with β close to 1, there are two solutions: mechanical impact on the flow and the measurement of β by a sensor in real-time with the transfer of this information to the signal processing program by the flowmeter. One of the obvious simple mechanical methods for suppressing pulsations is the installation of a large volume receiver in front of the flowmeter. However, at high flow rates, the volume of this receiver can also reach several tens of m^3^ or more, which is not always economically justified or possible. To control the effects of large β on measurement uncertainty, it is well advised to use a specially profiled flow contraction in the measurement region, which makes the velocity profile more uniform and significantly changes the structure of the boundary layer, where all the main resulting effects of the pulsations are observed. The second approach implies the use of the dynamic K coefficient by the data processing system of the flowmeter, which changes from the current value of β. To do this, the flowmeter must be supplemented with a high-speed pressure sensor that will measure the amplitude of the pulsations. In practice, in the vast majority of cases, flows with small β are encountered in the main systems. This is due to the fact that large values of β are associated with a strong overestimation of the flow velocity, which can cause acoustic noise, additional stresses on the structure, and cavitation in the case of fluid flow.

The analytical method proposed in present paper is universal and can be easily used for any other method of scanning the flow with ultrasonic beams, including for the further development of a scanning method that is insensitive to the presence of pulsations.

## Figures and Tables

**Table 1:** Basic acoustic path types for single- and multi-path meters.

Type	(a)	(b)	(c)	(d)	(e)	(f)
Number of chords	1	2	3	4	5	9
L	0	1/3 R	1/2 R	1/3 R 2/3 R	0 1/3 R 2/3 R	0 1/2 R

**Table 2:** Measurements of velocity profiles in pulsating flow.

He [11]			Papadopoulos [20]		
Regime	ω^+^	β	Regime	ω^+^	β
II	0.0039	0.20	V	0.043	0.16
III	0.0078	0.20	V	0.043	0.63
III	0.0194	0.20	V	0.087	0.63
III	0.0129	0.20	V	0.179	0.16
III	0.0128	0.20	V	0.176	0.32
III	0.0091	0.20	V	0.172	0.47
III	0.0091	0.47	V	0.172	0.63
IV	0.025	0.20	V	0.346	0.16
IV	0.0388	0.20	V	0.346	0.63

## Data Availability

Not applicable.
